# The shared genetic architecture of the TDP‐43 spectrum: Bridging clinical FTLD and neuropathological endophenotypes

**DOI:** 10.1002/alz.71943

**Published:** 2026-11-04

**Authors:** Maulikkumar Patel, Pawan Kumar, Daniel Western, Matthew Johnson, Menghan Liu, John Budde, Carlos Cruchaga, Cyril Pottier

**Affiliations:** ^1^ Department of Psychiatry Washington University School of Medicine St. Louis Missouri USA; ^2^ Neurogenomics and Informatics Center Washington University School of Medicine St. Louis Missouri USA; ^3^ Department of Neurology Washington University School of Medicine St. Louis Missouri USA; ^4^ Hope Center for Neurological Disorders Washington University School of Medicine St. Louis Missouri USA; ^5^ Knight Alzheimer Disease Research Center Washington University School of Medicine St. Louis Missouri USA

**Keywords:** frontotemporal lobar degeneration, *GRN*, genome‐wide association study, hippocampal sclerosis, limbic‐predominant age‐related TAR DNA‐binding protein 43 encephalopathy, *MAPT*, meta‐analyses, TAR DNA‐binding protein 43, *TMEM106B*

## Abstract

**INTRODUCTION:**

TAR DNA‐binding protein 43 (TDP‐43) proteinopathies encompass a spectrum of conditions including frontotemporal lobar degeneration with TDP‐43 inclusions (FTLD‐TDP), limbic‐predominant age‐related TDP‐43 encephalopathy (LATE‐NC), and hippocampal sclerosis (HS). FTLD‐TDP is further classified into several pathological subtypes (A, B, C, D, and E), which differ in the distribution and morphology of TDP‐43 inclusions. Clinical and neuropathological variability across FTLD, FTLD‐TDP, LATE‐NC, and HS has hindered the translatability of genetic discoveries between clinical and pathological disease categories. By leveraging their genetic convergence, we aimed to refine effect size estimates and uncover variants that influence the TDP‐43 spectrum.

**METHODS:**

We conducted an inverse variance weighted fixed‐effects meta‐analysis using METAL, combining neuropathological datasets (FTLD‐TDP subtypes: A [*N*_cases_ = 193], B [*N*_cases_ = 288], C [*N*_cases_ = 467], and controls [*N*_controls_ = 3153]), HS (*N*_cases_ = 751; *N*_controls_ = 6413), or LATE‐NC (*N*_cases_ = 1325; *N*_controls_ = 1787). Additionally, we performed a random‐effects meta‐analysis that included clinically diagnosed FTLD (*N*_cases_ = 4685; *N*_controls_ = 15,308) and neuropathological datasets. Moreover, we performed colocalization, fine‐mapping, and functional annotation analyses.

**RESULTS:**

Three loci reached genome‐wide significance. The *GRN* (rs5848) and *TMEM106B* (rs3807865) loci were associated with FTLD‐TDP (A and B), LATE‐NC, and HS.  Colocalization and fine‐mapping analyses supported genetic signals at *GRN* and *TMEM106B* loci, while functional annotation suggested that rs3807865 and rs3807866 influence regulatory activity and *TMEM106B* expression in glial cells. *MAPT* demonstrated association across all combinations of TDP‐43 proteinopathies (except FTLD‐TDP B and C) and clinical FTLD, specifically the H1c haplotype.

**DISCUSSION:**

Our results extend the known role of *GRN* and *TMEM106B* beyond FTLD‐TDP subtype A, while *MAPT* involvement suggests a role beyond traditional tauopathies.

## HIGHLIGHTS

- Meta‐analysis across clinical frontotemporal lobar degeneration (FTLD) and TAR DNA‐binding protein 43 (TDP‐43) proteinopathies identifies *MAPT*, *GRN*, and *TMEM106B* as key shared risk loci.
- *GRN* and *TMEM106B* associations extend beyond limbic‐predominant age‐related TDP‐43 encephalopathy, hippocampal sclerosis, and FTLD‐TDP subtype A to include FTLD‐TDP B.
- *MAPT* H1c is associated with clinical FTLD and FTLD‐TDP subtype A, suggesting subtype‐specific genetic effects across the TDP‐43 spectrum.

## 1 BACKGROUND

Neuropathologies featuring TAR DNA‐binding protein 43 (TDP‐43) inclusions in neuronal and glial cells have been widely reported, with frontotemporal lobar degeneration with TDP‐43 inclusions (FTLD‐TDP) being the most extensively studied form. FTLD‐TDP is classified into at least five subtypes—A, B, C, D and E^1^—based on the distribution and structure of TDP‐43 aggregates in the cortical layers of the brain. FTLD‐TDP manifests clinically into the FTLD syndrome characterized by gradual deterioration of the frontal and temporal regions of the brain, resulting in alterations in personality, changes in behavior, and language impairment.^2^ TDP‐43 positive cytoplasmic inclusions are present in up to 50% of FTLD patients.^3^, ^4^, ^5^ More recently, a new neurodegenerative entity called limbic‐predominant age‐related TDP‐43 encephalopathy neuropathologic change (LATE‐NC) has been identified, and involves distinct TDP‐43 neuropathological changes affecting the amygdala and hippocampus.^6^ The diagnostic criteria for LATE‐NC are based on the density of TDP‐43 immunoreactive neuronal cytoplasmic inclusions, with advanced stages indicating more widespread involvement in neocortical regions. Another condition associated with TDP‐43 proteinopathy is hippocampal sclerosis (HS), which commonly affects individuals over the age of 85 (≈ 10%) and for which up to 90% of cases are TDP‐43 positive.^7^, ^8^

Genetic studies, including genome‐wide association studies (GWASs) and whole‐genome sequencing (WGS), have identified loci associated with increased risk for FTLD‐TDP and related conditions, enhancing our understanding of these TDP‐43 proteinopathies’ etiology and progression. A large WGS study recently reported the *GRN* and *TMEM106B* loci associated with FTLD‐TDP,^9^ while Shade et al. showed that both of these loci were associated with LATE‐NC and HS.^10^ However, it did not always reached genome‐wide significance and additional studies are necessary to validate these associations.

The neuropathological and clinical variability of FTLD has limited the generalizability/biological relevance of genetic findings from clinically diagnosed FTLD and neuropathologically defined FTLD‐TDP, even though most clinically diagnosed FTLD patients will present with TDP‐43 pathology at autopsy.^11^, ^12^ For instance, *TNIP1* and *UNC13A*, associated with FTLD‐TDP, were not reported in the large clinical FTLD study,^12^ and similarly *MAPT* and *RPSA*/*MOBP*, associated with clinical FTLD, were not associated with FTLD‐TDP. On the other hand, the apolipoprotein E (*APOE*) locus was recently associated with clinical FTLD, which overlapped with findings in LATE‐NC.^10^, ^12^ Therefore, leveraging identified genetic loci across clinical FTLD, FTLD‐TDP, and related TDP‐43 proteinopathies such as LATE‐NC and HS provides an opportunity to bridge this gap. We hypothesized that this strategy could increase the power to detect TDP‐43–driven genetic risk factors, refine estimates of their effect sizes across endophenotypes, and highlight variants whose effects are consistent across both clinical and neuropathologically defined TDP‐43 proteinopathies. In this study, we therefore conducted a meta‐analysis integrating data from different FTLD and TDP‐43 proteinopathy studies. We comprehensively mapped the genetic landscape and enhanced our understanding of FTLD and TDP‐43 proteinopathies.

**RESEARCH IN CONTEXT**

1. **Systematic review**: The authors reviewed the most recent literature using traditional sources such as PubMed and Google Scholar. Clinical frontotemporal lobar degeneration (FTLD) and FTLD with TAR DNA‐binding protein 43 (TDP‐43) proteinopathies represent a complex overlapping spectrum of neurodegenerative conditions. Genetic studies including clinical FTLD and TDP‐43 proteinopathies have identified key risk genes such as *GRN*, *TMEM106B*, *MAPT*, and *MOBP*. The findings have enhanced our understanding of the genetic architecture and progression of these conditions.
2. **Interpretation**: The meta‐analyses presented in this paper integrate genetic data from clinical FTLD and TDP‐43 proteinopathies. This comprehensive approach has led to the identification of significant genetic associations, including the *MAPT*, *GRN*, and *TMEM106B* loci. The *GRN* and *TMEM106B* loci showed significant association across various TDP‐43 proteinopathies including FTLD‐TDP A and B subtypes. Moreover, this study highlights the potential role of *MAPT* as a modifier of TDP‐43 proteinopathies.
3. **Future directions**: Future research should focus on conducting further neuropathological studies to confirm the impacts of these genetic variants on disease progression and to validate these associations across diverse populations. These steps will deepen our understanding of genetic mechanisms underlying TDP‐43 proteinopathies and pave the way for therapeutic interventions.

## 2 METHODS

### 2.1 Study design

To identify potential studies, we searched the recent literature in PubMed and Google Scholar. We included any GWAS of clinical FTLD or neuropathological TDP‐43–related phenotypes for which genome‐wide summary statistics were available for harmonization and meta‐analysis. Studies were excluded if summary statistics were unavailable or if the phenotype was not relevant to the FTLD/TDP‐43 spectrum. In this study, we included both TDP‐43 proteinopathy studies and clinical FTLD (Table S1 in supporting information). The Pottier et al.^9^ study specifically targeted participants diagnosed with FTLD‐TDP (*n*_cases_ = 948; *n*_controls_ = 3153; *N* = 4101) and its subtypes, including FTLD‐TDP A (*n*_cases_ = 193), FTLD‐TDP B (*n*_cases_ = 288), and FTLD‐TDP C (*n*_cases_ = 467). From the Shade et al.^10^ study, we included pathological endophenotypes such as LATE‐NC (*n*_cases_ = 1325; *n*_controls_ = 1787; *N* = 3112) and HS (*n*_cases_ = 751; *n*_controls_ = 6413; *N* = 7164). In addition, as the majority of clinical cases of FTLD present with TDP‐43 neuropathology at autopsy, we included clinical FTLD from Manzoni et al.^12^ (*n*_cases_ = 4685; *n*_controls_ = 15308; *N* = 19993). The summary statistics from Pottier et al. were downloaded from the GWAS catalog (FTLD‐TDP: GCST90558311; FTLD‐TDP A: GCST90558312; FTLD‐TDP B: GCST90558313; FTLD‐TDP C: GCST90558314). Summary statistics from Shade et al. were obtained upon request to the corresponding author. Summary statistics from Manzoni et al. were downloaded from the University College London research data repository.^13^ The studies we used in our analysis include Pottier et al., which conducted WGS, and Manzoni et al. and Shade et al., which both performed single nucleotide polymorphism array–based GWASs.

### 2.2 Quality control and preprocessing

Rigorous quality control on raw data were previously performed as described.^9^, ^10^, ^12^ All GWAS summary statistics included in the meta‐analysis were derived from European ancestry–focused studies. In each GWAS study, population genetic ancestry was assessed using ancestry‐anchored principal components or admixture analyses. Accordingly, the meta‐analysis was conducted within a relatively homogeneous European ancestry population, reducing the likelihood of confounding due to population stratification. Genome‐wide summary statistics across all cohorts were harmonized and quality controlled using a standardized pipeline prior to downstream analyses. Variant coordinates were unified to the GRCh38 human reference genome build using chromosome, genomic position, and rsIDs. We used the Broad Institute liftover tool to convert variants originally based on GRCh37 to the GRCh38 reference genome to ensure uniformity.^14^ For bi‐allelic variants for which the effect and non‐effect alleles were harmonized between study datasets, and effect sizes (beta) were inverted to align effect estimates to a consistent reference allele. Variants with non‐complementary or mismatched alleles across studies (e.g., A/G vs. A/C) were excluded. To eliminate potential strand‐alignment errors, strand‐ambiguous variants (palindromic: A/T or C/G) with a minor or effect allele frequency between 0.45 and 0.55 were removed. Furthermore, lead variants identified in top loci were manually verified to confirm non‐palindromic status (Table S1).

### 2.3 Meta‐analysis approach

Inverse variance weighted meta‐analyses were conducted using METAL software,^15^ integrating both neuropathological and clinical datasets. The analyses were conducted in two stages. In the first stage, neuropathological datasets were combined across various combinations of FTLD‐TDP subtypes, LATE‐NC, and HS. Meta‐analysis models were selected based on the degree of phenotypic and methodological similarity across studies. For the neuropathological analysis, the two available cohorts were both based on brain autopsy and therefore showed high methodological homogeneity; a fixed‐effect model was used assuming a common underlying effect. In the second stage, clinical FTLD cohorts were combined with the neuropathological studies. Because these underlying diagnostic frameworks differ between these studies, we applied a random effect model to account for between‐study heterogeneity using Plink (v2.0). In addition to this unbiased genome‐wide approach, we specifically assessed sentinel variants of genome‐wide significant loci from clinical FTLD and neuropathological studies (*P* values [*P*] < 5×10^−08^). Note that the *APOE* variants failed quality controls in the Pottier et al.^9^ summary statistics and therefore could not be assessed.

### 2.4 *MAPT* sub‐haplotype analysis

To assess genetic signal for known *MAPT* haplotypes, we evaluated six variants tagging the *MAPT* haplotypes including rs1467967, rs242557 (a partial tag for the *H1c* sub‐haplotype), rs3785883, rs2471738, rs8070723 (a tag for the *H2* haplotype), and rs7521.^16^, ^17^, ^18^ The tag variants used to define haplotypes were selected according to linkage disequilibrium (LD) measures including *R* ^2^ and *D*′ (Table S2 in supporting information). Using summary statistics of FTLD‐TDP subtypes, HS, LATE‐NC, and clinical FTLD, we examined the association evidence at these six markers to infer whether the observed *MAPT* signals were consistent with specific haplotype backgrounds.

### 2.5 Colocalization analysis

Colocalization analysis between FTLD‐TDP (type A and type B) and LATE‐NC was performed at the *TMEM106B* and *GRN* loci using ± 250 kb regions centered on sentinel variants from the significant meta‐analyses. Colocalization was assessed using the coloc R package (v2.1).^19^ The posterior probability of hypothesis 4 (PP.H4) was used to assess evidence that the two traits share a common causal variant at the same locus, with higher PP.H4 values indicating stronger evidence for colocalization.

### 2.6 Fine‐mapping analysis

We performed fine mapping for each genome‐wide significant sentinel variant using SuSiE RSS, a Bayesian fine‐mapping method that integrates GWAS summary statistics with a LD correlation matrix to identify credible sets and estimates the posterior inclusion probabilities to identify likely causal variants. For the LD reference panel, we used 1000 Genomes project variant call data formatted as a Plink binary file and aligned to the GRCh38 genome build.^20^ We defined ± 500 kb genomic regions around the lead variant for LD calculation and 95% credible sets (i.e., each credible set has at least 95% probability of containing a true causal variant). Variants were prioritized based on their posterior inclusion probability (PIP), with higher PIP values indicating greater support for a variant being causal within a fine‐mapped credible set.

### 2.7 Functional analysis

AlphaGenome was used to predict the functional consequences of *TMEM106B* variants.^21^ Predictions were generated using a ± 500 kb sequence centered on the lead variant at the locus. Alzheimer's disease– and FTLD‐relevant brain tracks were identified directly from AlphaGenome output metadata using the keywords brain, cortex, cortical, frontal, temporal, hippocampus, hippocampal, entorhinal, prefrontal, microglia, microglial, neuron, neuronal, astrocyte, astrocytic, oligodendrocyte, oligodendroglia, glia, glial, cerebrum, cerebral, amygdala, substantia nigra, spinal cord. The evaluated modalities included RNA‐seq, CAGE, ATAC‐seq, DNase‐seq, histone‐modification ChIP‐seq, splice‐site probability, splice‐site usage, splice junction, and different histone‐track usage. GENCODE v46 annotations were used to display the variant relative to *TMEM106B*. The significant variants at the *TMEM106B* locus were queried in the SingleBrain portal to assess regulatory associations across different cell types.^22^

### 2.8 Generation of forest plots

To visually represent the effect sizes and confidence intervals of identified genetic associations, forest plots were generated using the forestplot R package (v3.1.7).

## 3 RESULTS

### 3.1 Study design

In this study, we aimed to dissect genetic risk loci associated with TDP‐43 proteinopathies using a two‐step approach (Figure 1). First, we focused on neuropathologically defined TDP‐43 proteinopathies and performed comprehensive fixed‐effect meta‐analyses of FTLD‐TDP and its subtypes with LATE‐NC and HS. Second, we leveraged the high expected proportion of TDP‐43 positive cases in clinical FTLD cohorts and performed random‐effects meta‐analyses to evaluate genetic associations across clinical FTLD and TDP‐43 proteinopathies.

**FIGURE 1:**
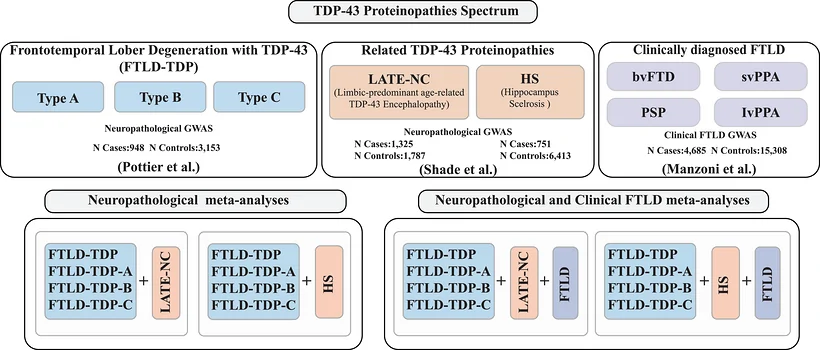
Schematic representation of TDP‐43 proteinopathies spectrum and study cohorts included. The figure depicts the neuropathological meta‐analysis study and the combined meta‐analysis of neuropathological and clinical FTLD studies. bvFTD, behavioral variant frontotemporal dementia; FTLD, frontotemporal lobar degeneration; GWAS, genome‐wide association study; HS, hippocampal sclerosis; LATE‐NC, limbic‐predominant age‐related TAR DNA‐binding protein 43 encephalopathy neuropathologic change; lvPPA, language variant primary progressive aphasia; PSP, progressive supranuclear palsy; svPPA, semantic variant primary progressive aphasia; TDP‐43, TAR DNA‐binding protein 43.

### 3.2 Meta‐analyses of TDP‐43 proteinopathies

We performed a meta‐analysis including datasets enriched in FTLD‐TDP patients from the study by Pottier et al. (FTLD‐TDP subtype A, FTLD‐TDP subtype B, and FTLD‐TDP subtype C) together with LATE‐NC data from Shade et al. (Table S1). The most significant locus was identified on chromosome 17, led by the rs5848 (*P*_meta:FTLD‐TDP+LATE‐NC_ = 5.96×10^−12^; beta (β)_meta:FTLD‐TDP+LATE‐NC_ = 0.280; standard error (SE) _meta:FTLD‐TDP+LATE‐NC_ = 0.041; effect allele = T; Figure 2A and Table 1; and Table S3 and Figure S1A in supporting information), which is located in the 3’UTR of the *GRN* gene. This variant was not previously reported as genome‐wide significant in the overall FTLD‐TDP group in the Pottier et al. study. However, because rs5848 previously reached genome‐wide significance in the FTLD‐TDP A subtype, we further meta‐analyzed each FTLD‐TDP subtype with LATE‐NC summary statistics. As expected, rs5848 was genome‐wide significant in FTLD‐TDP A and LATE‐NC meta‐analysis (*P*_meta:FTLD‐TDPA+LATE‐NC_ = 2.50 × 10^−12^, β_meta:FTLD‐TDP A+LATE‐NC_ = 0.356; SE_meta:FTLD‐TDP A+LATE‐NC_ = 0.050; effect allele = T; Figure 2A and Table 1; and Table S3 and Figure S1B). In line with this, colocalization analysis provided strong evidence for shared association signal with a 99.3% posterior probability of a shared causal variant (PP.H4). Interestingly, rs5848 was genome‐wide significant in FTLD‐TDP B and LATE‐NC meta‐analysis (*P*_meta:FTLD‐TDPB+LATE‐NC_ = 4.47×10^−9^, β_meta:FTLD‐TDPB+LATE‐NC_ = 0.288; SE_meta:FTLD‐TDPB+LATE‐NC_ = 0.049; effect allele = T; Figure 2A and Table 1; and Table S3 and Figure S1C). Colocalization analysis provided modest evidence for a shared causal signal between FTLD‐TDP B and LATE‐NC (PP.H4 = 65.2%). We identified a second genome‐wide significant signal at the *TMEM106B* locus driven by rs3807865 (*P*_meta:FTLD‐TDP+LATE‐NC_ = 4.31×10^−12^; β_meta:FTLD‐TDP+LATE‐NC_ = −0.267; SE_meta:FTLD‐TDP+LATE‐NC_ = 0.038; effect allele = A; reference allele = G; Figure 2A and Table 1; and Table S3 and Figure S1D). rs3807865 and the previously known protective variant rs1990622 at the *TMEM106B* locus are in strong LD (*D*′ = 0.96, *R^2^* = 0.90).^23^ Interestingly, rs3807865 also reached genome‐wide significance in the meta‐analysis of FTLD‐TDP B and LATE‐NC (*P*_meta:FTLD‐TDPB+LATE‐NC_ = 3.69×10^−12^; effect allele = A; Figure 2A; and Table S3 and Figure S1E). In the FTLD‐TDP A and LATE‐NC meta‐analysis, the *TMEM106B* locus was driven by rs6460900 (*P*_meta:FTLD‐TDPA+LATE‐NC_ = 8.45 × 10^−16^, β_meta:FTLD‐TDPA+LATE‐NC_ = −0.393; SE_meta:FTLD‐TDPA+LATE‐NC_ = 0.048; effect allele = A; Figure 2A and Table 1; and Table S3 and Figure S1F), while rs3807865 also reached genome‐wide significance (*P*_meta:FTLD‐TDPA+LATE‐NC_ = 1.84×10^−15^, β_meta:FTLD‐TDPA+LATE‐NC_ = −0.387; SE_meta:FTLD‐TDPA+LATE‐NC_ = 0.048; effect allele = A). rs6460900 and rs3807865 were in strong LD, with an *R^2^* of 0.94. Colocalization analysis provided strong evidence for shared association signal between FTLD‐TDP A and LATE‐NC (PP.H4 = 93.6%), whereas evidence for colocalization between FTLD‐TDP B and LATE‐NC was weak (PP.H4 = 26.9%), possibly due to limited statistical power. No signal at this locus was detected in FTLD‐TDP C.

**FIGURE 2:**
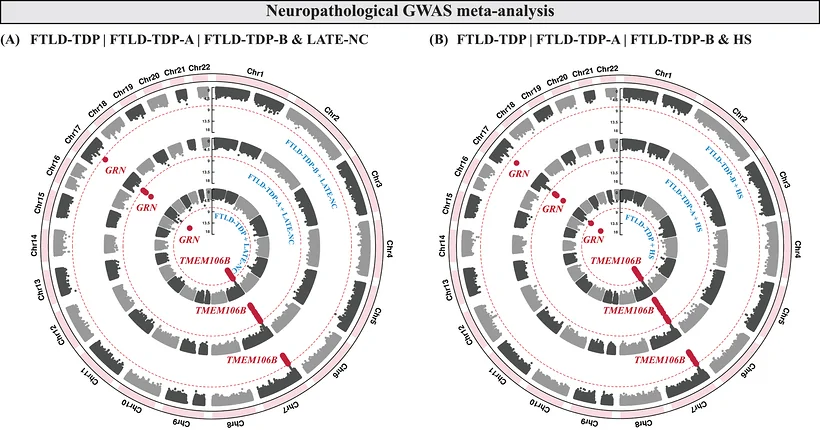
Meta‐analysis of neuropathological FTLD GWAS. The top‐most outer layer of the circle represents the chromosome number. The axis represents the ‐log10(*P* values) for each variant in the circular Manhattan plot. A, Circular Manhattan plot of the neuropathological GWAS meta‐analysis of FTLD‐TDP, FTLD‐TDP A, and FTLD‐TDP B with LATE‐NC. The inner circle shows the FTLD‐TDP and LATE‐NC meta‐analysis, the middle circle shows the FTLD‐TDP A and LATE‐NC meta‐analysis, and the outermost circle shows the FTLD‐TDP B and LATE‐NC meta‐analysis. B, Circular Manhattan plot of the neuropathological GWAS meta‐analysis of FTLD‐TDP, FTLD‐TDP A, and FTLD‐TDP B with HS. The inner circle shows the FTLD‐TDP and HS meta‐analysis, the middle circle shows the FTLD‐TDP A and HS meta‐analysis, and the outermost circle shows the FTLD‐TDP B and HS meta‐analysis. FTLD, frontotemporal lobar degeneration; GWAS, genome‐wide association study; HS, hippocampal sclerosis; LATE‐NC, limbic‐predominant age‐related TAR DNA‐binding protein 43 encephalopathy; TDP‐43, TAR DNA‐binding protein 43.

**TABLE 1:** Identification of genome‐wide significant hits from the meta‐analysis of pathological FTLD cohorts.

Pottier et al.^9^	Shade et al.^10^	Manzoni et al.^12^	rsID	Gene	Chr	Pos	A1	A2	BETA	SE	*P*
FTLD‐TDP	LATE‐NC	—	rs5848	*GRN*	17	44352876	T	C	0.280	0.041	5.96×10^−12^
FTLD‐TDP A	LATE‐NC	—	rs5848	*GRN*	17	44352876	T	C	0.356	0.050	2.50×10^−12^
FTLD‐TDP B	LATE‐NC	—	rs5848	*GRN*	17	44352876	T	C	0.288	0.049	4.47×10^−09^
FTLD‐TDP	LATE‐NC	—	rs3807865	*TMEM106B*	7	12210776	A	G	−0.267	0.038	4.31×10^−12^
FTLD‐TDP B	LATE‐NC	—	rs3807865	*TMEM106B*	7	12210776	A	G	−0.323	0.046	3.69×10^−12^
FTLD‐TDP A	LATE‐NC	—	rs6460900	*TMEM106B*	7	12213462	A	G	−0.393	0.048	8.45×10^−16^
FTLD‐TDP	HS	—	rs5848	*GRN*	17	44352876	T	C	0.305	0.041	1.75×10^−13^
FTLD‐TDP A	HS	—	rs5848	*GRN*	17	44352876	T	C	0.404	0.052	1.88×10^−14^
FTLD‐TDP B	HS	—	rs5848	*GRN*	17	44352876	T	C	0.328	0.051	1.09×10^−10^
FTLD‐TDP	HS	—	rs3807866	*TMEM106B*	7	12210752	A	G	−0.294	0.040	5.17×10^−13^
FTLD‐TDP B	HS	—	rs3807866	*TMEM106B*	7	12210752	A	G	−0.376	0.051	9.42×10^−14^
FTLD‐TDP A	HS	—	rs7805419	*TMEM106B*	7	12242825	C	T	−0.470	0.053	1.33×10^−18^
FTLD‐TDP	LATE‐NC	FTLD	rs2696557	*MAPT*	17	46269852	C	A	0.123	0.019	2.82×10^−11^
FTLD‐TDP A	LATE‐NC	FTLD	rs2696557	*MAPT*	17	46269852	C	A	0.128	0.019	1.87×10^−11^
FTLD‐TDP	HS	FTLD	rs199499	*MAPT*	17	46788132	T	C	−0.116	0.019	1.56×10^−09^

*Notes*: This table provides information on the meta‐analysis of the pathological FTLD cohort, including FTLD‐TDP and its subtypes with LATE or HS. Additionally, the meta‐analysis includes data combining clinical FTLD with pathological FTLD cohorts. Pottier et al.:^9^ pathological FTLD‐TDP and its subtype study; Shade et al.:^10^ pathological LATE‐NC and HS study; Manzoni et al.:^12^ clinical FTLD study. rsIDs = variant identification number in dbSNP; Chr = chromosome number; Pos = position on the chromosome; A1 = effect allele; A2 = other allele; BETA = effect size from the meta‐analysis; SE = variability of the estimated effect size; *P* = *P* value derived from meta‐analysis using fixed‐effect (pathological FTLD studies) or random effect (from clinical and pathological FTLD studies).

Abbreviations: FTLD, frontotemporal lobar degeneration; GWAS, genome‐wide association study; HS, hippocampal sclerosis; LATE‐NC, limbic‐predominant age‐related TAR DNA‐binding protein 43 encephalopathy; SE, standard error; TDP‐43, TAR DNA‐binding protein 43.

Similarly, both *GRN* and *TMEM106B* loci reached genome‐wide significance in the meta‐analysis of FTLD‐TDP and HS. The signal at the *GRN* locus was driven by rs5848 (*P*_meta:FTLD‐TDP+HS_ = 1.75×10^−13^; β_meta:FTLD‐TDP+HS_ = 0.305; SE_meta:FTLD‐TDP+HS_ = 0.041; effect allele = T; Figure 2B and Table 1; and Table S3 and Figure S2A in supporting information), and rs5848 reached genome‐wide significance in the meta‐analysis of FTLD‐TDP A and HS (*P*_meta:FTLD‐TDPA+HS_ = 1.88×10^−14^; β_meta:FTLD‐TDPA+HS_ = 0.404; SE_meta:FTLD‐TDPA+HS_ = 0.052; effect allele = T; Figure 2B and Table 1; and Table S3 and Figure S2B). The meta‐analysis including FTLD‐TDP B and HS also revealed rs5848 as genome‐wide significant (*P*_meta:FTLD‐TDPB+HS_ = 1.09×10^−10^; β_meta:FTLD‐TDPB+HS_ = 0.328; SE_meta:FTLD‐TDPB+HS_ = 0.051; effect allele = T; Figure 2B and Table 1; and Table S3 and Figure S2C).

In addition, we identified a genome‐wide significant signal at the *TMEM106B* locus driven by rs3807866 (*P*_meta:FTLD‐TDP+HS_ = 5.17×10^−13^; β_meta:FTLD‐TDP+HS_ = −0.294; SE_meta:FTLD‐TDP+HS_ = 0.040; effect allele = A; reference allele = G; Figure 2B and Table 1; and Table S3 and Figure S2D) for FTLD‐TDP. This variant reached genome‐wide significance in the meta‐analysis of FTLD‐TDP B and HS (*P*_meta:FTLD‐TDPB+HS_ = 9.42×10^−14^; *c*_meta:FTLD‐TDPB+HS_ = −0.376; SE_meta:FTLD‐TDPB+HS_ = 0.051; effect allele = A; Figure 2B and Table 1; and Table S3 and Figure S2E) and is in strong LD with the previously identified rs1990622 variant (*D*′ = 0.97, *R^2^* = 0.91). Moreover, the *TMEM106B* locus signal was driven by rs7805419 in the FTLD‐TDP A and HS meta‐analysis (*P*_meta:FTLD‐TDPA+HS_ = 1.33×10^−18^; β_meta:FTLD‐TDPA+HS_ = −0.470; SE_meta:FTLD‐TDPA+HS_ = 0.053; effect allele = C; Figure 2B and Table 1; and Table S3 and Figure S2F), with rs7805419 being in strong LD with rs1990622 (*D*′ = 1.0, *R^2^* = 0.98). The *TMEM106B* index variants identified across the FTLD‐TDP subtypes map to the same LD block, consistent with a shared association signal at this locus. No signal was detected from the FTLD‐TDP C subtype. Overall, our data suggests that the *GRN* and *TMEM106B* are associated with FTLD‐TDP A as well as FTLD‐TDP B.^9^

Because deciphering the causal variant at the *TMEM106B* is still an area of intense research, we performed fine mapping in this region. In the FTLD‐TDP and LATE‐NC analysis, the strongest fine‐mapping evidence was observed at the genome‐wide significant sentinel variant, rs3807865, which showed a PIP of 23%. In the FTLD‐TDP A and LATE‐NC analysis, the highest PIP was observed for rs2043539 at 9%, whereas in the FTLD‐TDP B and LATE‐NC analysis, rs3807865 showed the highest PIP at 10%. In the FTLD‐TDP and HS fine‐mapping analysis, the highest PIP was 20% for rs3807866. For FTLD‐TDP A and HS, the highest PIP was 7% for rs10281425, while for FTLD‐TDP B and HS, it was 8% for rs3807866. These genetic variants at the *TMEM106B* locus were in strong LD with the sentinel variant (*R^2^* > 0.80). These findings suggest that the lead genome‐wide significant variants remained among the most likely causal candidates within their respective loci, although the relatively low posterior probabilities likely reflect the high LD among correlated variants, which limits the ability to resolve a single causal variant in this region.

Furthermore, we identified several high‐ranking brain‐relevant regulatory predictions for candidate causal variants, including rs3807865 and rs3807866 at the *TMEM106B* locus. Even though modest, both rs3807865 and rs3807866 alternative alleles showed increased predicted chromatin accessibility in motor‐neuron and brain vascular endothelial cells. Despite this, H3K27ac predictions in astrocytes and spinal cord suggested that rs3807865 and rs3807866 alternative alleles weaken enhancer activity, potentially leading to reduced downstream *TMEM106B* expression (Figure 3 and Figure S3 in supporting information). Similarly, the alternative alleles of rs3807865 and rs3807866 reduced the promoter‐associated chromatin activity marker H3K4me3 in spinal‐cord astrocytes and brain microvascular endothelial cells. In line with this, single‐cell quantitative trait loci data showed that rs3807865 and rs3807866 alternative alleles were trending toward a reduced *TMEM106B* expression in astrocytes (*P* = 5.66×10^−2^; β = −0.039; *P* = 9.26×10^−2^; β = −0.034; respectively). Similarly, rs3807865 (*P* = 1.51×10^−2^; β = −0.051), rs3807866 (*P* = 1.74×10^−2^; β = −0.050), and rs6460900 (*P* = 1.55×10^−2^; β = −0.05) showed nominal associations with *TMEM106B* expression in oligodendrocytes, whereas rs7805419 showed a stronger association (*P* = 7.45×10^−5^; β = −0.06), supporting a potential cell type–specific regulatory role for *TMEM106B* variants.

**FIGURE 3:**
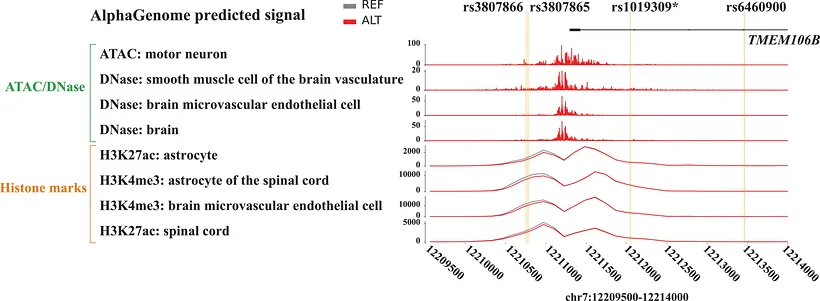
AlphaGenome‐predicted regulatory effects of variants at the *TMEM106B* locus. Reference‐allele predictions are shown in gray and alternate‐allele predictions in red across a 4‐kb interval at chr7:12,209,500‐12,214,000 (GRCh38). The figure displays brain‐relevant chromatin‐accessibility tracks, including motor‐neuron and brain vascular cell types; histone‐modification tracks in astrocytes, spinal cord, and brain microvascular endothelial cells. Vertical lines indicate rs3807866, rs3807865, rs1019309, and rs6460900 variants position. The largely overlapping REF and ALT profiles suggest modest allele‐dependent regulatory differences rather than a pronounced functional effect. * Represent variants previously reported by Gallagher et al.

### 3.3 Meta‐analyses of clinical FTLD datasets and TDP‐43 proteinopathies

Manzoni et al.^12^ conducted a comprehensive GWAS that incorporated a large number of clinically diagnosed FTLD participants. We first performed a meta‐analysis that included the FTLD‐TDP, LATE‐NC, and clinical FTLD datasets. We identified a single locus within the *MAPT* genomic region that achieved genome‐wide significance. rs2696557 was the most significant variant (*P*_meta:FTLD‐TDP+LATE‐NC+clinical‐FTLD_ = 2.82×10^−11^, β_meta:FTLD‐TDP+LATE‐NC+clinical‐FTLD_ = 0.123, SE_meta:FTLD‐TDP+LATE‐NC+clinical‐FTLD_ = 0.019, *I^2^* = 0.0; effect allele = C; Figure 4A and Table 1; and Table S4 and Figure S4A in supporting information). To assess whether the FTLD‐TDP signal was driven by a subtype, we performed meta‐analyses including the clinical FTLD, LATE‐NC, and FTLD‐TDP subtype studies. Interestingly, rs2696557 was nominally significant only in FTLD‐TDP A (*P*_FTLD‐TDPA_ = 1.55×10^−2^; β_FTLD‐TDPA_ = 0.350; SE_FTLD‐TDPA_ = 0.144; effect allele = C; Figure 4A and Table 1; and Table S4 and Figure S4B) as opposed to the other FTLD‐TDP subtypes, suggesting that the overall FTLD‐TDP signal is primarily driven by FTLD‐TDP A. The rs2696557 signal was also nominally significant in LATE‐NC (Table S4).

**FIGURE 4:**
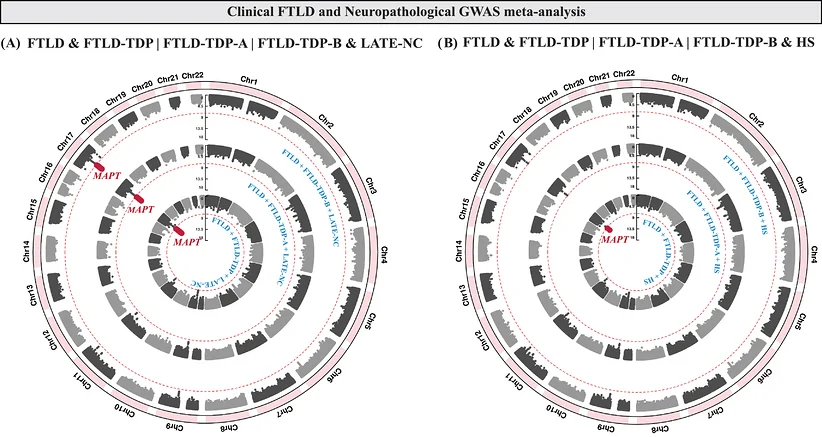
Meta‐analysis of clinical FTLD and neuropathological FTLD GWAS. The top‐most outer layer of the circle represents the chromosome number. The axis represents the ‐log10(*P* values) for each variant in the circular Manhattan plot. A, Circular Manhattan plot of the neuropathological GWAS and clinical FTLD meta‐analysis involving FTLD, FTLD‐TDP, and LATE‐NC. The inner circle represents the meta‐analysis of FTLD, FTLD‐TDP, and LATE‐NC; the middle circle represents the meta‐analysis of FTLD, FTLD‐TDP A, and LATE‐NC; and the outermost circle represents the meta‐analysis of FTLD, FTLD‐TDP B, and LATE‐NC. B, Circular Manhattan plot of the neuropathological GWAS and clinical FTLD meta‐analysis involving FTLD, FTLD‐TDP, and HS. The inner circle represents the meta‐analysis of FTLD, FTLD‐TDP, and HS; the middle circle represents the meta‐analysis of FTLD, FTLD‐TDP A, and HS; and the outermost circle represents the meta‐analysis of FTLD, FTLD‐TDP B, and HS. FTLD, frontotemporal lobar degeneration; GWAS, genome‐wide association study; HS, hippocampal sclerosis; LATE‐NC, limbic‐predominant age‐related TAR DNA‐binding protein 43 encephalopathy; TDP‐43, TAR DNA‐binding protein 43.

Given the well‐established haplotypic structure of the *MAPT* locus, we further evaluated the association of *H1* sub‐haplotypes with clinical FTLD and TDP‐43 proteinopathies. We observed a significant association signal at rs242557 (*P*_meta:FTLD‐TDPA+HS+clinical‐FTLD_ = 8.29×10^−4^; β_meta:FTLD‐TDPA+HS+clinical‐FTLD_ = 0.053, SE_meta:FTLD‐TDPA+HS+clinical‐FTLD_ = 0.016), a partial tag for the *MAPT H1c* sub‐haplotype, with FTLD‐TDP subtype A (*P*_FTLD‐TDPA_ = 8.95×10^−3^), clinical FTLD (*P*_clinical‐FTLD_ = 7.73×10^−3^), and LATE‐NC (*P*_LATE‐NC_ = 1.25×10^−1^).

Similar to the meta‐analysis with LATE‐NC, only the *MAPT* locus reached genome‐wide significance when including the HS study. rs199499 was the most significant variant (*P*_meta:clinical‐FTLD+FTLD‐TDP+HS_ = 1.56×10^−9^, β_meta:clinical‐FTLD+FTLD‐TDP+HS_ = −0.116, SE_meta:clinical‐FTLD+FTLD‐TDP+HS_ = 0.019, *I^2^* = 0.0; effect allele = T; Figure 4B and Table 1; and Table S4 and Figure S4C). The FTLD‐TDP signal was nominally significant in the FTLD‐TDP A subtype (*P*_FTLD‐TDPA_ = 3.70×10^−2^; β_FTLD‐TDPA_ = −0.306, SE_FTLD‐TDPA_ = 0.147). The rs2696557 variant was significant in LATE‐NC and showed a consistent direction of effect in HS. In addition, the *H1c* rs242557 tagging variant was also significant (*P*_meta:clinical‐FTLD+FTLD‐TDPA+HS_ = 4.99×10^−2^, β_meta:clinical‐FTLD+FTLD‐TDPA+HS_ = 0.104, SE_meta:clinical‐FTLD+FTLD‐TDPA+HS_ = 0.053; *P*_FTLD‐TDPA_ = 8.95×10^−3^, β_FTLD‐TDPA_ = 0.29, SE_FTLD‐TDPA_ = 1.110; *P*_FTLD_ = 7.73×10^−3^, β_clinical‐FTLD_ = 0.045, SE_clinical‐FTLD_ = 0.016; and *P*_HS_ = 7.34×10^−2^; β_HS_ = 0.105; SE_HS_ = 0.058).

Overall, our findings suggest that the *MAPT* locus is associated with multiple TDP‐43 proteinopathies and its *H1c* sub‐haplotype is primarily associated with clinical FTLD and the FTLD‐TDP A subtype.

### 3.4 Previously identified variants and meta‐analyses validation

Finally, we specifically assessed the other previously identified genome‐wide significant variants from all three studies at the *TNIP1*, *UNC13A*, and *RPSA*/*MOBP* loci. None of these variants and loci reached genome‐wide significance at the meta‐analysis stage (Table S5 in supporting information), suggesting that these variants are associated with a specific endophenotype.

## 4 DISCUSSION

In this study, we conducted an extensive meta‐analysis focusing on the genetic landscape of the TDP‐43 spectrum, leveraging data from both clinical FTLD and neuropathologically defined TDP‐43 proteinopathies. By integrating clinical FTLD data with TDP‐43 proteinopathies, we further delineated genetic association signals, particularly at the *MAPT* locus, which suggests that *MAPT* is implicated not only in clinically diagnosed FTLD but also in TDP‐43 proteinopathies. Our analyses confirmed and refined previously known association signals at the *GRN* and *TMEM106B* loci, further strengthening their roles in TDP‐43 proteinopathies. These findings bridge the gap between clinical presentations of FTLD and neuropathologically defined TDP‐43 proteinopathies and underscore the value of integrating genetic data from neuropathologically and clinically defined cohorts to enhance our understanding of these complex conditions.

We identified a strong association at the *MAPT* locus across our combined clinical and TDP‐43 proteinopathy datasets highlighting the critical role of this genomic region. Importantly, we revealed that the *MAPT* signal in FTLD‐TDP was driven by the FTLD‐TDP A subtype. This signal aligns with the previously reported association of the *MAPT H1* haplotype with an earlier age of onset in *GRN* mutation carriers, which consistently results with FTLD‐TDP A at autopsy.^24^ *MAPT* encodes the tau protein, which is known to aggregate in the brains of FTLD patients,^25^ including in a subset of patients with *MAPT* mutations.^26^ Accumulating neuropathological evidence reveals the presence of comorbid TDP‐43 pathology in some tauopathy patients, and vice versa, with distinct morphological characteristics and propagation patterns within the brain.^27^, ^28^, ^29^, ^30^ Of note, the *TMEM106B* locus has previously been linked to Braak neurofibrillary tangle stages.^10^ While further neuropathology studies are necessary, our findings strongly suggest that variants in the *MAPT* region may act as modifiers of TDP‐43 proteinopathy etiology.

Additionally, we reported the previously known *TMEM106B*‐associated locus with TDP‐43‐related proteinopathies. The association of variants at the *TMEM106B* locus with FTLD‐TDP type A was recently reported when collapsing common variants at the gene level (Pottier et al.^9^). We are now reporting on a genome‐wide significant association at the single variant level when combining FTLD‐TDP type A and LATE‐NC cases. Even though FTLD‐TDP type A and LATE‐NC are distinct entities in terms of clinical and regional disease burden,^31^, ^32^, ^33^ they share some histological features. Indeed, advanced LATE‐NC and FTLD‐TDP type A were recently reported as displaying similar densities of neuronal cytoplasmic inclusions and dystrophic neurites in amygdala and hippocampus.^33^, ^34^ In addition, both present with dense mesh‐like pattern in the hippocampus. On the other hand, we also report a genome‐wide significant signal when combining LATE‐NC and FTLD‐TDP type B. While FTLD‐TDP type A cases, like LATE‐NC, are associated with *GRN* and *TMEM106B* variants, FTLD‐TDP B cases have not been reported as such so far. The latter group of patients is usually associated with *C9ORF72* repeat expansions and more recently with *UNC13A* and *TNIP1* variants.^9^ We cannot, however, rule out that the FTLD‐TDP B signal from Pottier et al.^9^ originates from mixed FTLD‐TDP A and B pathologies.^35^, ^36^ Similarly, we also report that rs5848 at the *GRN* locus, which was previously thought to be FTLD‐TDP type A specific, reaches genome‐wide significance when combining FTLD‐TDP B subtype with LATE‐NC or HS. These findings suggest that the *TMEM106B* and *GRN* loci are not restricted to cases with FTLD‐TDP A and LATE‐NC pathology. In addition, this supports that *TMEM106B*‐ and *GRN*‐mediated pathways, such as endolysosomal regulation,^37^ may represent a broader mechanism across diverse TDP‐43 pathologies, rather than a subtype‐restricted risk factor.

To resolve whether a specific functional signal drives this broad association across FTLD‐TDP subtypes, we performed fine mapping at the *TMEM106B* locus and revealed multiple variants in strong LD with our lead signals, pointing to a complex underlying genetic mechanism. Historically, the *TMEM106B* locus has been characterized by a single LD block with as many as 104 genetic variants in strong LD (*R^2^* > 0.80) with the lead protective variant, rs1990622.^38^ However, a recent fine‐mapping effort has identified a finder structural composition of the locus.^39^ Salazar et al.^39^ reported that the *TMEM106B* locus contains multiple haplotypes associated with different neurodegeneration risks including Alzheimer's disease and FTLD. In our dataset, while our lead variant showed strong LD with the primary haplotypic background, it was not in LD with rs13237415, marker of the secondary independent LD block. Thus, our association signal did not capture the LD block2 signal described by Salazar et al. This finding adds to the growing body of evidence supporting the existence of a complex haplotypic structure at the *TMEM106B* locus.

Functional validations are, however, essential to identify the *TMEM106B* variant that directly influences gene regulation or protein function. In our study, rs3807865 and rs3807866 were associated with reduced H3K27ac‐marked regulatory activity at the *TMEM106B* locus in astrocytes, suggesting altered enhancer activity in glial cells at this locus. This finding is consistent with prior work showing that *TMEM106B* loss impairs lysosomal organization and remyelination in oligodendrocytes, and disrupts autophagosome maturation in astrocytes.^40^, ^41^ Moreover, rs7805419 is in near‐perfect LD with rs1990620, suggesting that it likely tags the same regulatory haplotype identified in previous studies, where the rs1990620 risk allele was shown to preferentially recruit CTCF transcription factor, strengthen long‐range chromatin interactions with the *TMEM106B* promoter, and increase *TMEM106B* expression in association with lysosomal abnormalities.^38^ Together, these findings suggest that *TMEM106B* regulatory variation as a plausible glial disease mechanism, linking non‐coding risk haplotypes to altered chromatin regulation, cell type–specific gene expression, and lysosomal dysfunction.

This work has several limitations. First, we relied on summary statistics rather than raw genotype data, which limited our ability to apply fully harmonized quality control and prevented complete assessment of some loci (e.g., *APOE*). Second, heterogeneity in case definitions and phenotyping across clinical FTLD and neuropathologically defined TDP‐43 proteinopathies may have diluted endophenotype‐specific signals and contributed to the absence of genome‐wide significance at some previously reported loci (e.g., *UNC13A*, *MOBP*). Finally, the majority of the participants from all studies were of European ancestry, restricting the generalizability of our findings to other populations.

Overall, our comprehensive meta‐analysis identified significant association signals at the *MAPT*, *GRN*, and *TMEM106B* loci, shedding light on their critical roles in the etiology and modulation of TDP‐43 proteinopathies. Importantly, our findings underscore the intricate genetic underpinnings linking tauopathies and TDP‐43 proteinopathies themselves. These insights not only enhance our understanding of the genetic architecture underlying FTLD and TDP‐43 proteinopathies but also highlight the importance of leveraging genetic data to bridge the gap between clinical and neuropathological diagnoses. Ultimately, our findings pave the way for improved genetic risk assessments and hold promise for the development of targeted therapeutic strategies to mitigate the burden of these devastating diseases.

## AUTHOR CONTRIBUTIONS

Cyril Pottier and Maulikkumar Patel designed and oversaw the study. Maulikkumar Patel and Cyril Pottier were responsible for acquisition of data. Maulikkumar Patel, Pawan Kumar, Matthew Johnson, John Budde, Menghan Liu, and Cyril Pottier did the analysis or interpretation of the data. Maulikkumar Patel, Cyril Pottier, and Carlos Cruchaga did the statistical analysis of the data. Maulikkumar Patel, Carlos Cruchaga, and Cyril Pottier drafted the manuscript content. Maulikkumar Patel, Pawan Kumar, Matthew Johnson, Menghan Liu, John Budde, Carlos Cruchaga, and Cyril Pottier revised the manuscript for content. Cyril Pottier did the study supervision and coordination. Maulikkumar Patel, Pawan Kumar, Matthew Johnson, John Budde, Daniel Western, Menghan Liu, Carlos Cruchaga, and Cyril Pottier provided intellectual input. All authors contributed to and reviewed the final version of the manuscript.

## CONFLICT OF INTEREST STATEMENT

The authors declare no conflicts of interest. Author disclosures are available in the Supporting Information.

## CONSENT STATEMENT

Informed consent was not necessary for this study.

## Supporting information



Supporting Information

Supporting Information
