# Supplementary material for: The shared genetic architecture of the TDP‐43 spectrum: Bridging clinical FTLD and neuropathological endophenotypes
Source: Alzheimers Dement. 2026 Nov 4;22(11):e71943. doi: 10.1002/alz.71943 (PMC13736556; doi:10.1002/alz.71943)
Supplement: Supplementary file 2 [file ALZ-22-e71943-s001.docx]

Table of Contents

[**Supplementary Tables**	2](#_Toc239929283)

[Table S1: Demographic information of clinical FTLD and pathological FTLD characteristics across three different GWAS studies.	2](#_Toc239929284)

[Table S2: LD statistics of *MAPT* subhaplotypes tagged variants.	3](#_Toc239929285)

[Table S3: Identification of genome-wide significant hits from the meta-analysis of pathological FTLD cohorts	4](#_Toc239929286)

[Table S4: Identification of genome-wide significant hits from the meta-analysis of clinical and pathological FTLD cohorts	5](#_Toc239929287)

[Table S5: Top-hits from prior GWAS identified in clinical and pathological GWAS meta-analyses.	6](#_Toc239929288)

[**Supplementary Figures**	7](#_Toc239929289)

[Supplementary Figure S1: Meta-analyses of FTLD-TDP and its subtypes with LATE-NC.	7](#_Toc239929290)

[Supplementary Figure S2: Meta-analyses of FTLD-TDP and its subtypes with HS.	8](#_Toc239929291)

[Supplementary Figure S3: AlphaGenome-predicted functional effects of variants across *TMEM106B* locus.	9](#_Toc239929292)

[Supplementary Figure S4: Meta-analyses of TDP-43 proteinopathies (FTLD-TDP subtypes, LATE-NC or HS) and clinical FTLD.	10](#_Toc239929293)

# **Supplementary Tables**

Table S1: Demographic information of clinical FTLD and pathological FTLD characteristics across three different GWAS studies.

GWAS: GWAS from the previously published study; Study: summary statistics of each of their study; N participants: Number of total participants; N cases: Number of cases in each study; N controls: Number of controls in each study; N variants: Number of variants in each study

| **GWAS** | **Study** | **N**  **participants** | **N**  **cases** | **N**  **controls** | **N**  **variants** |
| --- | --- | --- | --- | --- | --- |
| Pottier et al.(1) | FTLD-TDP | 4,101 | 948 | 3,153 | 6,515,179 |
|  | FTLD-TDP A | 3,346 | 193 | 3,153 | 6,454,973 |
|  | FTLD-TDP B | 3,441 | 288 | 3,153 | 6,731,423 |
|  | FTLD-TDP C | 3,620 | 467 | 3,153 | 6,580,127 |
| Shade et al.(2) | LATE-NC | 3,112 | 1,325 | 1,787 | 8,958,601 |
|  | HS | 7,164 | 751 | 6,413 | 8,937,945 |
| Manzoni et al.(3) | Clinical FTLD | 19,993 | 4,685 | 15,308 | 6,338,702 |

Table S2: LD statistics of *MAPT* subhaplotypes tagged variants.

rsID of SNP1: reference SNP identifier for variant 1; rsID of SNP2: reference SNP identifier for variant 2; R²: LD correlation measure; D′: LD measure reflecting recombination history

| **rsID of SNP1** | **rsID of SNP2** | **R^2^** | **D'** |
| --- | --- | --- | --- |
| rs1467967 | rs242557 | 0.017 | 0.255 |
| rs1467967 | rs3785883 | 0.002 | 0.072 |
| rs1467967 | rs2471738 | 0.021 | 0.407 |
| rs1467967 | rs8070723 | 0.132 | 1.000 |
| rs1467967 | rs7521 | 0.146 | 0.494 |
| rs242557 | rs3785883 | 0.000 | 0.005 |
| rs242557 | rs2471738 | 0.185 | 0.608 |
| rs242557 | rs8070723 | 0.198 | 1.000 |
| rs242557 | rs7521 | 0.000 | 0.035 |
| rs3785883 | rs2471738 | 0.000 | 0.020 |
| rs3785883 | rs8070723 | 0.060 | 1.000 |
| rs3785883 | rs7521 | 0.003 | 0.108 |
| rs2471738 | rs8070723 | 0.099 | 1.000 |
| rs2471738 | rs7521 | 0.151 | 0.820 |
| rs8070723 | rs7521 | 0.221 | 1.000 |

Table S3: Identification of genome-wide significant hits from the meta-analysis of pathological FTLD cohorts

This table represents the genome-wide significant hits identified from the meta-analysis of FTLD-TDP and its subtypes with LATE-NC or HS.

Pottier et al.(1): Pathological FTLD-TDP and its subtype study; Shade et al.(2):Pathological LATE-NC and HS study; rsIDs: variant identification number in dbSNP; Chr: chromosome number; Pos: Position on the chromosome; A1: effect allele; A2: other allele; BETA: effect size from the meta-analysis; SE: variability of the estimated effect size; Meta P: P-value derived from meta-analysis; Direction: Direction of the effect observed; Pottier et al.: BETA, SE and P from pottier et al. study; Shade et al.: BETA, SE and P from Shade et al. study;

|  |  |  |  |  |  |  |  | **Meta** | | | **Pottier et al.(1)** | | | **Shade et al.(2)** | | |
| --- | --- | --- | --- | --- | --- | --- | --- | --- | --- | --- | --- | --- | --- | --- | --- | --- |
| **Pottier**  **et al.(1)** | **Shade**  **et al.(2)** | **rsIDs** | **Gene** | **Chr** | **Pos** | **A1** | **A2** | **BETA** | **SE** | **P** | **BETA** | **SE** | **P** | **BETA** | **SE** | **P** |
| FTLD-TDP | LATE-NC | rs5848 | *GRN* | 17 | 44352876 | T | C | 0.280 | 0.041 | 5.96×10^-12^ | 0.280 | 0.057 | 9.22×10^-7^ | 0.278 | 0.057 | 1.29×10^-6^ |
| FTLD-TDP A | LATE-NC | rs5848 | *GRN* | 17 | 44352876 | T | C | 0.356 | 0.050 | 2.50×10^-12^ | 0.635 | 0.108 | 5.57×10^-9^ | 0.278 | 0.057 | 1.29×10^-6^ |
| FTLD-TDP B | LATE-NC | rs5848 | *GRN* | 17 | 44352876 | T | C | 0.288 | 0.049 | 4.47×10^-9^ | 0.315 | 0.094 | 8.68×10^-4^ | 0.278 | 0.057 | 1.29×10^-6^ |
| FTLD-TDP | LATE-NC | rs3807865 | *TMEM106B* | 7 | 12210776 | A | G | -0.267 | 0.038 | 4.31×10^-12^ | -0.185 | 0.055 | 9.03×10^-4^ | -0.342 | 0.053 | 1.49×10^-10^ |
| FTLD-TDP B | LATE-NC | rs3807865 | *TMEM106B* | 7 | 12210776 | A | G | -0.323 | 0.046 | 3.69×10^-12^ | -0.263 | 0.094 | 5.44×10^-3^ | -0.342 | 0.053 | 1.49×10^-10^ |
| FTLD-TDP A | LATE-NC | rs6460900 | *TMEM106B* | 7 | 12213462 | A | G | -0.393 | 0.048 | 8.45×10^-16^ | -0.598 | 0.118 | 4.35×10^-7^ | -0.350 | 0.053 | 5.85×10^-11^ |
| FTLD-TDP | HS | rs5848 | *GRN* | 17 | 44352876 | T | C | 0.305 | 0.041 | 1.75×10^-13^ | 0.280 | 0.057 | 9.22×10^-7^ | 0.332 | 0.060 | 3.23×10^-8^ |
| FTLD-TDP A | HS | rs5848 | *GRN* | 17 | 44352876 | T | C | 0.279 | 0.040 | 1.88×10^-12^ | 0.635 | 0.108 | 5.57×10^-9^ | 0.332 | 0.060 | 3.23×10^-8^ |
| FTLD-TDP B | HS | rs5848 | *GRN* | 17 | 44352876 | T | C | 0.328 | 0.051 | 1.09×10^-10^ | 0.315 | 0.094 | 8.68×10^-4^ | 0.332 | 0.060 | 3.23×10^-8^ |
| FTLD-TDP | HS | rs3807866 | *TMEM106B* | 7 | 12210752 | A | G | -0.294 | 0.040 | 5.17×10^-13^ | -0.182 | 0.055 | 1.08×10^-3^ | -0.423 | 0.059 | 1.41×10^-12^ |
| FTLD-TDP B | HS | rs3807866 | *TMEM106B* | 7 | 12210752 | A | G | -0.376 | 0.051 | 9.42×10^-14^ | -0.259 | 0.094 | 6.18×10^-3^ | -0.423 | 0.059 | 1.41×10^-12^ |
| FTLD-TDP A | HS | rs7805419 | *TMEM106B* | 7 | 12242825 | C | T | -0.470 | 0.053 | 1.33×10^-18^ | -0.612 | 0.119 | 3.08×10^-7^ | -0.434 | 0.059 | 3.18×10^-13^ |

Table S4: Identification of genome-wide significant hits from the meta-analysis of clinical and pathological FTLD cohorts

This table represents the genome-wide significant hits identified from the meta-analysis of clinical FTLD and TDP-43 proteinopathies including FTLD-TDP and its subtypes and LATE-NC or HS.

Pottier et al.(1): Pathological FTLD-TDP and its subtype study; Shade et al.(2): Pathological LATE-NC and HS study; Manzoni et al.(3): Clinical FTLD study; rsIDs: variant identification number in dbSNP; Chr: chromosome number; Pos: Position on the chromosome; A1: effect allele; A2: other allele; BETA: effect size from the meta-analysis; SE: variability of the estimated effect size; Random P: P-value derived from meta-analysis using random effect; Direction: Direction of the effect observed; Het I^2^ : variation across study due to heterogeneity; Pottier et al.: BETA, SE and P from pottier et al. study; Shade et al.: BETA, SE and P from Shade et al. study; Manzoni et al.: BETA, SE and P from Manzoni et al. study.

|  |  | |  |  |  |  |  |  |  | **Meta** | | | **Pottier et al.(1)** | | | **Shade et al**.**(2)** | | | **Manzoni et al.(3)** | | |
| --- | --- | --- | --- | --- | --- | --- | --- | --- | --- | --- | --- | --- | --- | --- | --- | --- | --- | --- | --- | --- | --- |
| **Pottier**  **et al.(1)** | **Shade**  **et al.(2)** | **Manzoni et al.(3)** | | **rsID** | **Chr** | **pos** | **A1** | **A2** | **Gene** | **BETA** | **SE** | **P** | **BETA** | **SE** | **P** | **BETA** | **SE** | **P** | **BETA** | **SE** | **P** |
| FTLD-TDP | LATE-NC | | FTLD | rs2696557 | 17 | 46269852 | A | C | *MAPT* | 0.123 | 0.019 | 2.82×10^-11^ | 0.111 | 0.067 | 1.00×10^-1^ | 0.184 | 0.064 | 4.06×10^-3^ | 0.118 | 0.020 | 4.57×10^-9^ |
| FTLD-TDP A | LATE-NC | | FTLD | rs2696557 | 17 | 46269852 | A | C | *MAPT* | 0.128 | 0.019 | 1.87×10^-11^ | 0.350 | 0.144 | 1.55×10^-2^ | 0.184 | 0.064 | 4.06×10^-3^ | 0.118 | 0.020 | 4.57×10^-9^ |
| FTLD-TDP | HS | | FTLD | rs199499 | 17 | 46788132 | T | C | *MAPT* | -0.116 | 0.019 | 1.56×10^-9^ | -0.139 | 0.070 | 4.74×10^-2^ | -0.210 | 0.074 | 4.50×10^-3^ | -0.107 | 0.020 | 2.78×10^-7^ |
| FTLD-TDP A | HS | | FTLD | rs199499 | 17 | 46788132 | T | C | *MAPT* | -0.118 | 0.019 | 1.82×10^-3^ | -0.306 | 0.147 | 3.70×10^-2^ | -0.210 | 0.074 | 4.50×10^-3^ | -0.107 | 0.020 | 2.78×10^-7^ |
| FTLD-TDP B | HS | | FTLD | rs199499 | 17 | 46788132 | T | C | *MAPT* | -0.118 | 0.019 | 2.80×10^-4^ | -0.235 | 0.120 | 5.06×10^-2^ | -0.210 | 0.074 | 4.50×10^-3^ | -0.107 | 0.020 | 2.78×10^-7^ |

Table S5: Top-hits from prior GWAS identified in clinical and pathological GWAS meta-analyses.

Pottier et al.(1): Pathological FTLD-TDP and its subtype study; Shade et al.(2): Pathological LATE-NC and HS study; Manzoni et al.(3): Clinical FTLD study; rsIDs: variant identification number in dbSNP; Chr: chromosome number; Pos: Position on the chromosome; A1: effect allele; A2: other allele; BETA: effect size from the meta-analysis; SE: variability of the estimated effect size; Meta P: P derived from meta-analysis; Direction: Direction of the effect observed; Random P: P derived frim the random-effect meta-analysis; I^2^ : heterogeneity value across different studies

| **Pottier**  **et al.(1)** | **Shade**  **et al.(2)** | **Manzoni**  **et al.(3)** | **rsIDs** | **Gene** | **Chr** | **Pos** | **A1** | **A2** | **BETA** | **SE** | **P** | **Direction** | **I^2^** |
| --- | --- | --- | --- | --- | --- | --- | --- | --- | --- | --- | --- | --- | --- |
| FTLD-TDP | LATE-NC | FTD | rs17650901 | *MAPT* | 17 | 45962325 | G | A | 0.117 | 0.018 | 1.67×10^-10^ | +++ | 0.0 |
| FTLD-TDP | LATE-NC | FTD | rs1009966 | *RPSA/MOBP* | 3 | 39432100 | G | A | 0.071 | 0.015 | 1.38×10^-1^ | -++ | 57.91 |
| FTLD-TDP A | LATE-NC | FTD | rs17650901 | *MAPT* | 17 | 45962325 | G | A | 0.122 | 0.019 | 4.15×10^-4^ | +++ | 36.13 |
| FTLD-TDP A | LATE-NC | FTD | rs1009966 | *RPSA/MOBP* | 3 | 39432100 | G | A | 0.078 | 0.016 | 4.31×10^-7^ | -++ | 0.0 |
| FTLD-TDP B | LATE-NC | FTD | rs17650901 | *MAPT* | 17 | 45962325 | G | A | 0.117 | 0.019 | 4.77×10^-10^ | +++ | 0.0 |
| FTLD-TDP B | LATE-NC | FTD | rs1009966 | *RPSA/MOBP* | 3 | 39432100 | G | A | 0.072 | 0.015 | 7.08×10^-1^ | -++ | 77.49 |
| FTLD-TDP C | LATE-NC | FTD | rs17650901 | *MAPT* | 17 | 45962325 | G | A | 0.112 | 0.019 | 4.02×10^-5^ | -++ | 15.95 |
| FTLD-TDP C | LATE-NC | FTD | rs1009966 | *RPSA/MOBP* | 3 | 39432100 | G | A | 0.079 | 0.015 | 2.23×10^-7^ | +++ | - |
| FTLD-TDP | HS | - | rs5848 | *GRN* | 17 | 44352876 | T | C | 0.305 | 0.041 | 1.75×10^-13^ | ++ | - |
| FTLD-TDP | HS | - | rs871269 | *TNIP1* | 5 | 151052827 | T | C | -0.208 | 0.043 | 1.38×10^-6^ | -- | - |
| FTLD-TDP A | HS | - | rs5848 | *GRN* | 17 | 44352876 | T | C | 0.404 | 0.053 | 1.88×10^-14^ | ++ | - |
| FTLD-TDP A | HS | - | rs17650901 | *MAPT* | 17 | 45962325 | G | A | 0.318 | 0.065 | 1.05×10^-6^ | ++ | - |
| FTLD-TDP B | HS | - | rs5848 | *GRN* | 17 | 44352876 | T | C | 0.328 | 0.051 | 1.10×10^-10^ | ++ | - |
| FTLD-TDP C | HS | - | rs5848 | *GRN* | 17 | 44352876 | T | C | 0.234 | 0.048 | 1.05×10^-6^ | ++ | - |
| FTLD-TDP | LATE-NC | - | rs5848 | *GRN* | 17 | 44352876 | T | C | 0.280 | 0.041 | 5.47×10^-12^ | ++ | - |
| FTLD-TDP | LATE-NC | - | rs8111424 | *UNC13A* | 19 | 17640336 | G | A | -0.205 | 0.043 | 1.94×10^-6^ | -- | - |
| FTLD-TDP | LATE-NC | - | rs1990622 | *TMEM106B* | 7 | 12244161 | G | A | -0.249 | 0.038 | 9.82×10^-11^ | -- | - |
| FTLD-TDP | LATE-NC | - | rs2043539 | *TMEM106B* | 7 | 12214254 | G | A | -0.258 | 0.038 | 1.93×10^-11^ | -- | - |
| FTLD-TDP A | LATE-NC | - | rs5848 | *GRN* | 17 | 44352876 | T | C | 0.357 | 0.051 | 2.50×10^-12^ | ++ | - |
| FTLD-TDP A | LATE-NC | - | rs1990622 | *TMEM106B* | 7 | 12244161 | G | A | -0.383 | 0.048 | 3.59×10^-15^ | -- | - |
| FTLD-TDP A | LATE-NC | - | rs2043539 | *TMEM106B* | 7 | 12214254 | G | A | -0.390 | 0.048 | 1.09×10^-15^ | -- | - |
| FTLD-TDP B | LATE-NC | - | rs5848 | *GRN* | 17 | 44352876 | T | C | 0.289 | 0.049 | 4.47×10^-9^ | ++ | - |
| FTLD-TDP B | LATE-NC | - | rs1990622 | *TMEM106B* | 7 | 12244161 | G | A | -0.308 | 0.046 | 2,80×10^-11^ | -- | - |
| FTLD-TDP B | LATE-NC | - | rs2043539 | *TMEM106B* | 7 | 12214254 | G | A | -0.322 | 0.046 | 4.19×10^-12^ | -- | - |
| FTLD-TDP C | LATE-NC | - | rs1990622 | *TMEM106B* | 7 | 12244161 | G | A | -0.204 | 0.043 | 2.32×10^-6^ | -- | - |
| FTLD-TDP C | LATE-NC | - | rs2043539 | *TMEM106B* | 7 | 12214254 | G | A | -0.218 | 0.043 | 4.78×10^-7^ | -- | - |

# **Supplementary Figures**

Supplementary Figure S1: Meta-analyses of FTLD-TDP and its subtypes with LATE-NC. Forest plots summarizing study-specific and pooled meta-analyses effect size estimates for associations between FTLD-TDP subtypes and LATE-NC, for *GRN* loci (rs5848; panels A–C) and *TMEM106B* loci (rs3807865, panels D–E; rs6460900, panel F). Circle represents the individual study-wise effect sizes with 95% confidence intervals shown by horizontal lines. Diamonds represent the pooled effect sizes, with their width corresponding to the 95% confidence intervals.

Supplementary Figure S2: Meta-analyses of FTLD-TDP and its subtypes with HS. Forest plots summarizing study-specific and pooled meta-analyses effect size estimates for associations between FTLD-TDP subtypes and HS, for *GRN* loci (rs5848; panels A–C) and *TMEM106B* loci (rs3807866, panels D–E; rs7805419, panel F). Circle represents the individual study-wise effect sizes with 95% confidence intervals shown by horizontal lines. Diamonds represent the pooled effect sizes, with their width corresponding to the 95% confidence intervals.

Supplementary Figure S3: AlphaGenome-predicted functional effects of variants across *TMEM106B* locus. Reference-allele predictions are shown in gray and alternate-allele predictions in red across chr7:12,206,269–12,248,367 (GRCh38). Brain-relevant tracks include frontal and dorsolateral prefrontal cortex RNA-seq, amygdala CAGE, motor-neuron ATAC-seq, brain and brain-vascular DNase-seq, astrocytic and spinal-cord H3K27ac/H3K4me3, brain microvascular endothelial CTCF binding, and splice-site predictions. Vertical lines indicate rs3807866, rs3807865, rs1019309, rs2043539, and rs1990622 variants position. The broad regional view provides genomic context for the localized regulatory signals near rs3807865 and facilitates comparison of REF and ALT predictions across multiple molecular modalities. * Represents variants previously reported by Gallagher et al. and Salazar et al.

Supplementary Figure S4: Meta-analyses of TDP-43 proteinopathies (FTLD-TDP subtypes, LATE-NC or HS) and clinical FTLD. Forest plots summarizing study-specific and pooled meta-analyses effect size estimates for associations between FTLD-TDP, FTLD-TDP A with LATE-NC and clinical FTLD for *MAPT* (rs2696557, panels A–B), and for the effect sizes estimates of *MAPT* loci for FTLD-TDP, HS and clinical FTLD (rs199499, panel C). Circle represents the individual study-wise effect sizes with 95% confidence intervals shown by horizontal lines. Diamonds represent the pooled effect sizes, with their width corresponding to the 95% confidence intervals.

**References:**

1.	Pottier C, Kucukali F, Baker M, Batzler A, Jenkins GD, van Blitterswijk M, et al. Deciphering distinct genetic risk factors for FTLD-TDP pathological subtypes via whole-genome sequencing. Nat Commun. 2025;16(1):3914.

2.	Shade LMP, Katsumata Y, Abner EL, Aung KZ, Claas SA, Qiao Q, et al. GWAS of multiple neuropathology endophenotypes identifies new risk loci and provides insights into the genetic risk of dementia. Nat Genet. 2024;56(11):2407-21.

3.	Manzoni C, Kia DA, Ferrari R, Leonenko G, Costa B, Saba V, et al. Genome-wide analyses reveal a potential role for the MAPT, MOBP, and APOE loci in sporadic frontotemporal dementia. Am J Hum Genet. 2024;111(7):1316-29.
